# Outcomes after transcatheter aortic valve replacement in older patients

**DOI:** 10.1007/s00059-020-04986-0

**Published:** 2020-10-07

**Authors:** Kaffer Kara, Axel Kloppe, Aydan Ewers, Leif Bösche, Assem Aweimer, Habib Erdogan, Dominik Schöne, Fabian Schiedat, Nikolaos Patsalis, Peter Lukas Haldenwang, Justus Thomas Strauch, Andreas Mügge, Polykarpos C. Patsalis

**Affiliations:** 1 Heart Center Bergmannsheil, Department of Cardiology and Angiology, University Hospital Bergmannsheil, Ruhr University Bochum, Bürkle-de-la-Camp-Platz 1, 44789 Bochum, Germany; 2 Heart Center Bergmannsheil, Department of Cardiothoracic Surgery, University Hospital Bergmannsheil, Ruhr University Bochum, Bochum, Germany; 3 Department of Cardiology, Agaplesion General Hospital Hagen, Hagen, Germany; 4 Department of Cardiology, Angiology and Intensive Care Medicine, Philipps University Marburg, Marburg, Germany

**Keywords:** Aortic stenosis, Balloon valvuloplasty, Direct implantation, Aged, Outcome, Aortenstenose, Ballonvalvuloplastie, Direkte Implantation, Senioren, Outcome

## Abstract

**Background:**

The prevalence of aortic valve stenosis is increasing due to the continuously growing geriatric population. Data on procedural success and mortality of very old patients are sparse, raising the question of when this population may be deemed as “too old even for transcatheter aortic valve replacement (TAVR).” We, therefore, sought to evaluate the influence of age on outcome after TAVR and the impact of direct implantation.

**Methods:**

The data of 394 consecutive patients undergoing TF-TAVR were analyzed. Patients were divided into four age groups: ≤75 (group 1, *n* = 28), 76–80 (group 2, *n* = 107), 81–85 (group 3, *n* = 148), and >85 (group 4, *n* = 111) years. Direct implantation was performed when possible according to current recommendations. Survival was evaluated by Kaplan–Meier analysis.

**Results:**

Mortality at 30 days and 1 year was not significantly different between the four age groups (3.6 vs. 6.7 vs. 5.4 vs. 2.7% and 7.6 vs. 17 vs. 14.5 vs. 13%m respectively, log-rank *p* = 0.59). Direct implantation without balloon aortic valvuloplasty was more frequently performed on patients aged >85 vs. ≤85 years (33.3 vs. 14.1%, *p* < 0.001). the incidence of procedural complications frequently associated with advanced age (stroke, vascular complications) was not significantly increased in group 4.

**Conclusion:**

Outcome after TF-TAVR is comparable among different age cohorts, even in very old patients. Direct implantation simplifies the procedure and could therefore play a role in reducing the incidence of peri-interventional complications in patients of advanced age.

## Abstract (de)

**Hintergrund:**

Die Prävalenz der Aortenklappenstenose steigt durch eine kontinuierlich wachsende geriatrische Bevölkerung. Daten über prozeduralen Erfolg sowie Mortalität von sehr alten Patienten sind gering, sodass sich die Frage stellt, wann diese Population „als bereits zu alt“ für einen Transkatheter-Aortenklappenersatz (TAVR) anzusehen wäre. Ziel dieser Studie war es, den Einfluss der direkten Implantation auf die Ergebnisse nach transfemoraler (TF-)TAVR bei Patienten in fortgeschrittenem Alter zu evaluieren.

**Methoden:**

Dazu wurden die Daten von 394 konsekutiven Patienten nach TF-TAVR ermittelt. Die Patienten wurden in 4 Altersgruppen eingeteilt: ≤75 Jahre (Gruppe 1, *n* = 28), 76–80 Jahre (Gruppe 2, *n* = 107), 81–85 Jahre (Gruppe 3, *n* = 148) und >85 Jahre (Gruppe 4, *n* = 111). Sofern es die aktuellen Empfehlungen erlaubten, wurde eine direkte Implantation durchgeführt. Das Überleben wurde mittels Kaplan–Meier-Analyse evaluiert.

**Ergebnisse:**

Zwischen den 4 Altersgruppen waren keine signifikanten Unterschiede der Mortalität nach 30 Tagen und nach einem Jahr zu verzeichnen (entsprechend 3,6 vs. 6,7 vs. 5,4 vs. 2,7 % und 7,6 vs. 17 vs. 14,5 vs. 13 %; *p* = 0,59 für Log-Rank-Test). Eine direkte Implantation ohne Ballonvalvuloplastie wurde bei Patienten >85 Jahre häufiger durchgeführt als ≤85 Jahre (33,3 vs. 14,1 %; *p* < 0,001). Die Inzidenz von häufig mit fortgeschrittenem Patientenalter assoziierten prozeduralen Komplikationen (Schlaganfall, vaskuläre Komplikationen) war in der Gruppe 4 nicht signifikant erhöht.

**Schlussfolgerung:**

In verschiedenen Alterskollektiven zeigen sich vergleichbare Ergebnisse nach TF-TAVR, dies gilt sogar für Patienten sehr hohen Alters. Die direkte Implantation kann die Prozedur vereinfachen und zu einer konsekutiven Reduktion der Inzidenz von periprozeduralen Komplikationen bei Patienten mit fortgeschrittenem Alter führen.

Transfemoral transcatheter aortic valve replacement (TF-TAVR) has evolved to the standard of care for patients with severe symptomatic aortic valve stenosis who are at prohibitive, high, and even intermediate risk for surgical aortic valve replacement [1–4]. The prevalence of aortic valve stenosis is increasing due to the continuously growing geriatric population [4–6]. However, data on procedural success and mortality of very old patients are sparse, raising the question of when is this population “too old even for TAVR.” In addition, the incidence of some TAVR-associated complications (stroke, vascular complications) appears to be more frequent in elderly patients [6]. Nevertheless, current data show that avoidance of preparatory balloon aortic valvuloplasty (BAV) can be associated with procedural simplification and thus lower complication rates [7]. In addition, the influence of direct TAVR without preparatory BAV on the incidence of TAVR-associated complications in the very old and more fragile population needs to be investigated. The purpose of the present study was therefore to evaluate the influence of age on outcome after TF-TAVR and the possible impact of direct implantation in very old patients.

## Patients and methods

### Patient population

Data from 394 consecutive high-risk patients with symptomatic aortic valve stenosis who underwent transfemoral (TF) TAVR in our center using the Medtronic Corevalve Evolut R (MER) or Medtronic Evolut Pro (MEP; Medtronic Inc., Minneapolis, MN, USA; *n* = 44), the Edwards SAPIEN 3 (ES3; Edwards Lifesciences Inc., Irvine, CA, USA; *n* = 258), the Symetis ACURATE neo (SAN; Boston Scientific Corporation, Natick, MA, USA; *n* = 71), the Direct flow (DF; Direct Flow Medical, Santa Rosa California, USA; *n* = 19), and the Portico (Abbot Vascular, Illinois, USA; *n* = 2) bioprostheses were analyzed retrospectively. Patients were divided into four age groups: age ≤75 (group 1, *n* = 28), 76–80 (group 2, *n* = 107), 81–85 (group 3, *n* = 148), and >85 years (group 4, *n* = 111). Due to the increased frailty, direct implantation without preparatory BAV was more frequently performed on patients aged over 85 years (group 4) due to procedure simplification. The decision for TAVR was made by an interdisciplinary heart team [1, 8, 9]. All TAVR procedures were performed according to standard techniques and guidelines [8–11].

### Paravalvular leakage

Residual paravalvular leakage (PVL) was graded qualitatively according to the Sellers criteria [12]. In order to assist on-table decision-making, the amount of regurgitating contrast medium during supra-aortic angiography after final device deployment defined PVL severity [12, 13]: absent 0/4, mild 1/4, moderate 2/4, moderate-to-severe 3/4, and severe 4/4 [12, 13]. In addition, simultaneous left ventricular (LV) and aortic pressures were recorded at 50 mm/s and averaged over three representative cardiac cycles after the procedure [12, 13]. For quantitative evaluation of PVL severity, the pressure gradient between diastolic aortic and left ventricular end-diastolic pressure (∆P_DAP–LVEDP_) was assessed [12]. A ∆P_DAP–LVEDP_ ≤18 mm Hg has been previously associated with increased mortality, especially in cases of relevant PVL after TAVR [12].

### Endpoint

The primary endpoint was all-cause mortality at 30 days and 1 year according to the Valve Academic Research Consortium (VARC II) definitions [10]. The incidence of other TAVR-associated complications, with a focus on stroke and vascular complications, and THV performance were further evaluated. The follow up period was 1 year.

### Postinterventional protocol

After TAVR, patients were transferred for 24 h to an intensive care unit for postinterventional monitoring. Besides the clinical examination, electrocardiogram, body temperature check, and chest x‑ray, all blood parameters that had already been determined at the initial examination were assessed again. Follow-up examinations were performed 30 days and 1 year after discharge.

### Statistical analysis

Categorical data are presented as frequencies and percentages; continuous variables are presented as means and standard deviation. The normal distribution of the variables was tested by the Shapiro–Wilk test (*p*≥0.1). Comparisons were made with two-sided χ^2^ tests or two-sided Fisher’s exact tests for categorical variables and one-way ANOVA for continuous variables, using Bonferroni correction for multiple testing. An ANOVA and *t *test were used to compare normally distributed variables and the Mann–Whitney test to compare the other non-normally distributed variables between the four age groups. Statistical significance was set at *p*<0.05. Survival analyses for the four age groups were performed using the Kaplan–Meier method, with patients censored as of the last date known alive. All statistical analyses were performed using SPSS (version 17.0, SPSS, Chicago, IL, USA). The authors had full access to the data and take full responsibility for their integrity. All authors have read and agree to the manuscript as written.

## Results

### Baseline and procedural characteristics

Our study cohort represents a typical TF-TAVR patient population deemed as high risk for open heart surgery with symptomatic aortic stenosis (aortic valve area 0.7 ± 0.2 cm^2^, transvalvular gradient 47.0 ± 16.0 mm Hg). Patients in group 4 had a significantly higher EuroSCORE compared with the younger groups (12.7 ± 10.0 vs. 11.5 ± 7.5 vs. 14.8. ± 9.9 vs. 20.9 ± 12.2, respectively, *p* < 0.001). In addition, patients in group 4 were, as expected, significantly older and had significantly less weight and height (Table 1a). The aortic valve area of the very old patients in group 4 was significantly smaller compared with the other age groups.

**Table 1:** Baseline (A) and postprocedural (B) characteristics

	Overall (*n* = 394)	Group 1: ≤75 (*n* = 28)	Group 2: ≤76–80 (*n* = 107)	Group 3: 81–85 (*n* = 148)	Group 4: >85 (*n* = 111)	*p*
**A**						
Age, years	82.6 ± 4.9	72.8 ± 3.3	78.6 ± 1.4	83.0 ± 1.4	88.3 ± 2.4	<0.001
Male gender	184 (46.7)	19 (67.9)	54 (50.5)	69 (46.6)	42 (37.8)	0.027
Weight, kg	75.7 ± 15.4	84.2 ± 13.7	79.7 ± 16.0	75.7 ± 12.8	69.8 ± 16.3	<0.001
Height, cm	167.2 ± 10.1	172.0 ± 8.0	167.5 ± 8.5	168.2 ± 8.6	164.4 ± 13.0	<0.001
Logistic EuroSCORE, %	15.5 ± 10.7	12.7 ± 10.0	11.5 ± 7.5	14.8 ± 9.9	20.9 ± 12.2	<0.001
Aortic valve area, cm^2^	0.7 ± 0.2	0.7 ± 0.3	0.7 ± 0.2	0.7 ± 0.2	0.6 ± 0.2	0.046
Mean transvalvular PG before TAVR, mm Hg	47.0 ± 16.0	45.8 ± 17.4	45.5 ± 16.8	48.3 ± 16.8	46.7 ± 16.9	0.41
LVEF, %	54.1 ± 11.1	48.6 ± 14.6	53.7 ± 10.5	54.8 ± 10.9	55.1 ± 10.5	0.16
CAD	215 (54.6)	15 (53.6)	55 (51.4)	80 (54.1)	65 (58.6)	0.76
Prior MI	49 (12.4)	4 (14.3)	12 (11.2)	15 (10.1)	18 (16.2)	0.47
Prior PCI	127 (32.2)	11(39.3)	35 (32.7)	42 (28.4)	39 (35.1)	0.54
Prior heart surgery	32 (8.1)	4 (14.3)	12 (11.2)	10 (6.8)	6 (5.4)	0.2
PVD	40 (10.2)	2 (7.1)	13 (12.1)	11 (7.4)	14 (12.6)	0.44
**B**						
Mean transvalvular PG after TAVR, mm Hg	10.4 ± 4.4	10.7 ± 4.3	10.4 ± 4.5	10.5 ± 4.2	10.2 ± 4.6	0.88
Vascular complications (major)	18 (4.7)	2 (7.1)	19 (17.8)	20 (13.5)	16 (14.4)	0.56
Vascular complications (minor)	23 (6.0)	0 (0)	8 (7.6)	8 (5.6)	7(6.5)	0.58
Stroke (disabling)	5 (1.3)	0 (0)	2 (1.9)	3 (2.0)	0 (0)	0.49
Stroke (non-disabling)	6 (1.5)	1 (3.6)	1 (0.9)	2 (1.4)	2 (1.8)	0.64

Values are mean ± SD, *n* (%)

*CAD* coronary artery disease, *LVEF* left ventricular ejection fraction, *MI* myocardial infarction, *PCI* percutaneous coronary intervention, *PVD* peripheral vascular disease, *PG* pressure gradient

There were no other significant differences in baseline and postprocedural characteristics between the four age groups (Table 1a, b).

### Mortality and peri-interventional complications

**Fig. 1:**
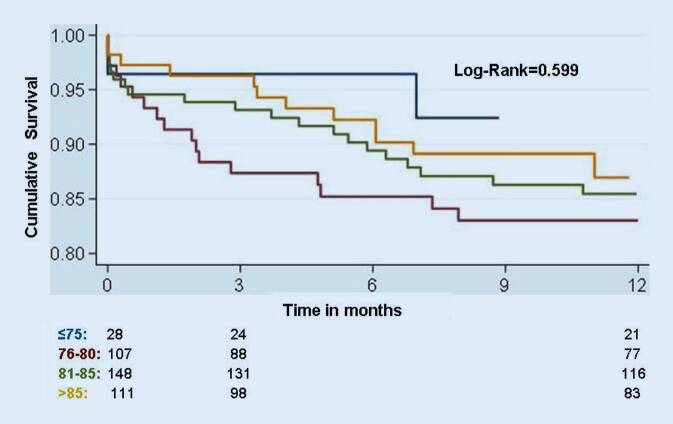
Cumulative survival of the four age groups. Mortality at 30 days and 1 year was not significantly different between the four age groups (log-rank = 0.59)

**Fig. 2:**
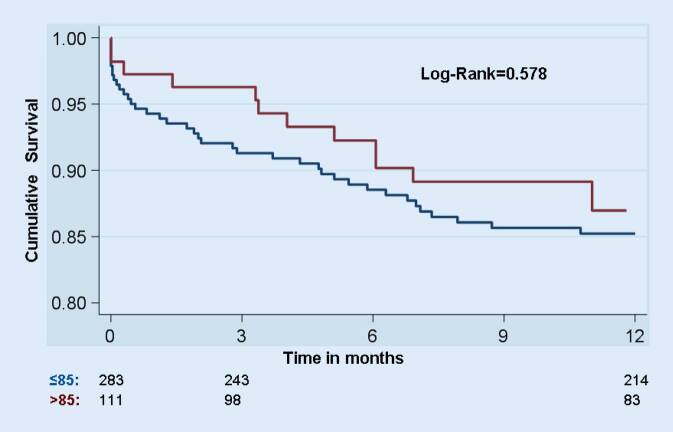
Cumulative survival of patients aged >85 vs. ≤85. Patients aged >85 showed a statistically nonsignificant trend toward a better outcome than patients aged ≤85 (log-rank = 0.578)

Mortality at 30 days and 1 year was not significantly different between the four age groups (3.6 vs. 6.7 vs. 5.4 vs. 2.7% and 7.6 vs. 17 vs. 14.5 vs. 13%, respectively, log-rank *p* = 0.59; Fig. 1). Direct implantation without balloon aortic valvuloplasty was more frequently performed on patients aged >85 vs. ≤85 years (33.3 vs. 14.1%, *p* < 0.001). The incidence of procedural complications frequently associated with advanced age (stroke, vascular complications) was not significantly increased in group 4 (Table 1b). In a further analysis, patients aged ≤85 and >85 years were compared. Patients aged >85 showed a statistically nonsignificant trend toward a better outcome than patients aged ≤85 (log-rank= 0.578; Fig. 2).

### Paravalvular leakage after TAVR

The angiographic assessment of postprocedural PVL revealed a similar distribution of PVL severity after TF-TAVR (Table 2a) between patients aged ≤85 and >85 years. Severe PVL did not occur in any of our study patients. Hemodynamic assessment of PVL severity showed a similar incidence in the pressure difference of ∆P_DAP–LVEDP_ <18 mm Hg between the two groups (Table 2b).

**Table 2:** Assessment of paravalvular leakage severity

PVL	≤85 years (*n* = 279)	>85 years (*n* = 111)	*p*
**A**			
Absent (0/4)	180 (64.5%)	78 (70.3%)	–
Trace or mild (1/4)	70 (25.1%)	27 (24.3%)	–
Moderate (2/4)	29 (10.4%)	6 (5.4%)	0.27
Moderate-to-severe (3/4)	0 (0%)	0 (0%)	–
Severe (4/4)	0 (0%)	0 (0%)	–
**B**			
∆P_DAP–LVEDP_ <18 mm Hg	37 (13.7)	10 (9.1)	0.30

The distribution of postprocedural paravalvular leakage (PVL) after transfemoral transcatheter aortic valve replacement (TF-TAVR; A) and invasive hemodynamics (B). Values are *n* (%)

### Impact of THV type on direct implantation and mortality

Direct implantation was performed significantly more frequently with the ES3 bioprosthesis (*n* = 54 patients, 70.1%) than the MER or MEP bioprosthesis (*n* = 23 patients, 29.9%; *p* = 0.015). Preparatory BAV was always performed when the other THVs were used.

Mortality at 30 days and 1 year was not significantly different between the five THV groups (ES3, MER or MEP, SAN, DF, Portico; 4.0 vs. 6.8 vs. 7.1 vs. 5.3% vs. 0% and 15.4 vs. 9.1 vs. 16.5 vs. 5.3% vs. 0%, respectively, log-rank *p* = 0.665).

## Discussion

The present study demonstrates that TF-TAVR can be safely performed on the very old patient population with similar outcomes to younger patients. Procedural simplification might lead to lower complication rates after TAVR especially in the very fragile population with increased age. In this analysis, the prevalence of stroke and minor or major vascular complications that have been previously associated with increased age was not significantly increased in the patients over 85 years undergoing TF-TAVR [5, 7, 14, 15]. In addition, age did not significantly impact the outcomes of patients undergoing TF-TAVR.

### Impact of age on outcome

Randomized control studies have shown that age was not an independent determinant of 1‑year mortality [4]. Nevertheless, comorbidities may influence the outcome of younger patients, which can explain the similar survival rates between different age groups [4]. On the other hand, there are data showing an association between increasing age and in-hospital mortality after TAVR [4, 6, 14, 15]. In a similar analysis, there was a trend toward higher 30-day and 6‑month mortality in patients older than 90 years old; however, the difference was not significant [16]. In the present study, contrary to what was expected based on the significantly higher operative risk and fragility of very old patients, patients aged >85 years had a better outcome than did patients aged 76–80 and 81–85 years, most likely as a result of more serious comorbid conditions limiting life expectancy.

### Incidence of age-associated complications and impact of increased fragility

The geriatric population has grown, leading to an increased number of patients undergoing TAVR. Taking this epidemiological fact into consideration, improvements in transcatheter technology and increased operator experience leading to further simplification of the procedure are key to achieving the best result in such a fragile population [6, 7].

According to recent data, the incidence of periprocedural complications defined by the Valve Academic Research Consortium may be similar between the different age groups [4]. Nevertheless, stroke and vascular complications have been observed more frequently in TAVR patients of increased age [5, 7, 14, 15]. The degree of vascular calcification and frailty may play an important role in these observations; however, this remains hypothetical and needs further investigation [6].

In this study, the incidence of stroke and vascular complications was not higher in the very old patient group undergoing TF-TAVR. This analysis shows that TF-TAVR can be performed with similar good procedural results in the continuously increasing very old patient population.

Interestingly, very old patients of group 4 had a significantly lower weight and height. Low BMI has been associated with significantly worse outcome and is considered an independent predictor of mortality [4]. In addition, as expected due to the increased age, the logistic EuroSCORE was significantly higher in the group of very old patients. Therefore, based on current data, the very old population of group 4 had an unfavorable starting position compared with the younger patients. This analysis showed that despite significantly increased fragility proved by quantitative parameters of pre-interventional risk stratification (height, weight, logistic EuroSCORE), TF-TAVR can be safely performed with very good outcomes even in very old high-risk patients. Moreover, this study demonstrated non-significant trends towards lower mortality rates in group 4.

A current analysis has shown that modern direct TAVR, performed without the use of preparatory BAV, leads to lower complication rates probably due to the simplification of the procedure [7]. Over 5000 patients were analyzed, showing significant advantages for the patients undergoing direct TAVR (quicker procedures, lower amounts of contrast and radiation, lower tamponade rates; [7]). In this study, direct implantation without preparatory BAV was more frequently performed on patients aged >85 years. In our hands, direct TF-TAVR may provide a simple method to decrease such “age-associated” complications not only by reducing unnecessary exchange maneuvers in the aortic arch and the left ventricle but also at the same time by avoiding the additional rapid pacing needed for the BAV. The positive impact of direct TF-TAVR in terms of procedure simplification could partly explain the similarly low rates of “age-associated” complications in the very old patient group compared with the younger patients, although this remains speculative and requires further investigation.

Balloon aortic valvuloplasty can improve annular sizing, facilitate the delivery system passing through the stenotic native valve, and is supposed to optimize valve expansion [7, 17]. Nevertheless, BAV has been related to hemodynamic instability, acute aortic regurgitation, renal failure, increased incidence of stroke, and pacemaker implantation [7, 18–20].

### Limitations

Our data are derived from a retrospective analysis of consecutive patients and not from a prospective, randomized trial. We therefore cannot exclude that part of the observed, not necessarily expected, benefit in group 4 is due to a learning curve and not specifically to the technique of direct implantation. In this study, direct TF-TAVR was intermittently performed on very old patients according to a procedure simplification. Further investigation is necessary to evaluate whether the positive impact of direct TF-TAVR in patients with advanced age remains if direct implantation is routinely and evenly used in all age groups. However, direct-TAVR is not always applicable and preparatory BAV can still be a necessity for many patients [17]. Therefore, the conclusion on the impact of direct TAVR remains hypothetical.

## Conclusion

Outcome after transfemoral–transcatheter aortic valve replacement is comparable among different age cohorts, even in very old patients. Direct implantation may be key for further reduction of peri-interventional complications especially in patients of advanced age. Age per se is not a strong parameter for pre-interventional risk stratification.

## Abbreviations

BAV: Balloon aortic valvuloplasty
DAP: Diastolic aortic pressure
∆P_DAP–LVEDP_: Pressure gradient between DAP and LVEDP
LVEDP: Left ventricular end-diastolic pressure
PVL: Paravalvular leakage
THV: Transcatheter heart valve
